# Growth performances, gastrointestinal epithelium and bacteria responses of Yellow-feathered chickens to kudzu-leaf flavonoids supplement

**DOI:** 10.1186/s13568-021-01288-4

**Published:** 2021-09-03

**Authors:** Fuguang Xue, Gen Wan, Yunsen Xiao, Chuanbin Chen, Mingren Qu, Lanjiao Xu

**Affiliations:** 1 Jiangxi Province Key Laboratory of Animal Nutrition/Engineering Research Center of Feed Development, Jiangxi Agricultural University, Nanchang, 330045 Jiangxi China; 2 Nanchang Key Laboratory of Animal Health and Safety Production, Jiangxi Agricultural University, Nanchang, 330045 Jiangxi China

**Keywords:** Yellow-feathered broilers, Kudzu-leaf flavonoids, Anti-oxidant, Antibiotic alternative, Gastrointestinal health

## Abstract

The objective of this study was to investigate the effects of replacing antibiotics with Kudzu-leaf flavonoids (KLF) on the growth performances, gut epithelial development, and gastrointestinal bacteria diversities of Yellow-feathered broilers. For this purpose, total of 216 1-day-old male Yellow-feathered chickens with the similar birth weight (31.0  ±  1.0 g) were randomly divided into 3 treatments: the control treatment (CON), the kudzu-leaf flavonoids supplement treatment (KLF), and the antibiotics supplement treatment (AGP). All birds were provided with a 56 d-feeding procedure, followed by the measurement of production performances, immune organs, blood anti-oxidant parameters, intestine epithelium development, and cecal microbiota. Results showed the feed conversion ratio significantly decreased after KLF supplement compared with CON (*P * <  0.05). KLF supplement partly promoted the anti-oxidant capacity on account of the increased activity of Superoxide dismutase (SOD) and the decrease content of malondialdehyde (MDA). Further, as referred to the gastrointestinal development and bacteria, ratio of villus/crypt significantly increased of ileum in KLF treatment (*P*  <  0.05) while a significant promition of bacterial diversity and partial representative probiotic bacteria (*P * <  0.05) after KLF supplementation. Moreover, correlation analysis indicated that probitics including *Bifidobacterium, Butyricimonas, Lactobacillus and Streptococcus* positively correlated with production performances*.* In conclusion, KLF supplement may promote feed efficiency and benefit the gastrointestinal health through improving gut bacterial diversity and probiotic bacteria. The KLF might be applied as a proper antibiotic alternative.

**Supplementary Information:**

The online version contains supplementary material available at 10.1186/s13568-021-01288-4.

## Introduction

The feed antibiotics were popularly used in the poultry industry to maintain animal health and improve growth performance. Despite the enhancement for broiler production over the past decades (Chapman and Johnson 2002), antibiotics now threated both animal and human health through causing the inhibition of protein synthesis (macrolides and tetracyclines), the interference on nucleic acid synthesis (fluoroquinolones and rifampin), the inhibition of a metabolic pathway, and superbugs (Claudie et al. 2009; Wasch et al. 1998). Therefore, seeking proper antibiotic alternatives, including plant essential oils, probiotics and antimicrobial peptides have been in process in the past few years (SHIM et al. 2010; Wang et al. 2015).

Flavonoids, which was mainly extracted in plant leaves, drew great attention in antibiotic alternative investigation on account of their broad-spectrum antimicrobial capacity and antioxidant activity (Brenes et al. 2008; Fernandez et al. 2002). Proper amount of flavonoids supplement to the feeding diet improved the growth performances of broilers. Besides, flavonoids were also proved to proliferate the intestinal microflora in broilers, which enriched the intestinal microbiota diversity, and further promoted nutrient digestibility and absorption (Pan and Yu 2014; Zhang 2018). However, whether flavonoids supplement affected the gut epithelial development, and the interactive effects between bacteria and intestine on the growth performance are still unclear. Therefore, in the present study, flavonoid extracted from Kudzu-leaf was applied to investigate the effects on growth performance, gut epithelial development, and cecal bacteria of Yellow-feathered broilers. We hypothesized that flavonoid may improve gastrointestinal bacteria community and intestinal epithelium development to promote the growth of broilers.

## Materials and methods

### Experimental design and birds feeding procedure

Total of 216 1-day-old female Yellow-feathered chickens with the similar birth weight (31.0  ±  1.0 g) were randomly divided into 3 treatments: the control treatment (CON), the kudzu-leaf flavonoids supplement treatment (KLF), and the antibioticsupplement treatment (AGP), respectively. KLF was acquired from Huawave Biotech Co. Ltd., Xi’an, China, and the purity was 75%, and the supplement amount of KLF is 0.06% of the total diets based on our pre-treatment. Antibiotics were acquired from Huamengjinhe industrial Co. Ltd., Inner Mongolia, China, with 15% Aureomycin content. Each treatment contained 6 replicates, with 12 broilers in each replicate. All birds were provided a 56-day-long feeding process, which was divided into two phases (day 0–28, as starter phase, day 29–56, as finisher phase). The diets used in the starter and finisher phases were shown in Table 1. The room temperature was maintained at 35 °C for the first week and then reduced by 2 °C each week until reaching 24 °C. The lighting schedule was 23 h light and 1 h dark during the experiment period. Feed and water were provided ad libitum throughout the experiment. Birds were vaccinated against Newcastle disease and infectious bronchitis according to the requirement of normal immunization procedures.

**Table 1:** Composition and nutrition level of the experimental diets for Yellow-feathered chickens

Ingredient	Starter phase	Finisher phase
Corn	59.7	60.4
Soy oil	1.45	2.98
SBM, CP 43%	34.6	32.68
L- LysHCL, (98%)	0.17	0.18
DL-Met	0.24	0.23
CaCO_3_	1.2	1
Calcium hydrophosphate (2 water) DCP	1.86	1.8
Salt	0.4	0.4
Choline HCl (50%)	0.15	0.1
Primix Vitamin^a^	0.03	0.03
Primix mineral^b^	0.2	0.2
Total	100	100
ME/(kcal/kg)	2950	3020
CP	21	20
Ca	1.01	0.9
P	0.45	0.43
dLys	1.15	1.1
dMet	0.5	0.48
dCys	0.29	0.28
dM + C	0.86	0.82

^a^Vitamin content: VA12000IU/kg; VD33000IU/kg; VE7.5 IU/kg; VK31.50 mg/kg; VB1 0.6 mg/kg; VB2 4.8 mg/kg; VB6 1.8 mg/kg; VB12 10 mg/kg; Folic acid 0.15 mg/kg; niacinamide 30 mg/kg; pantothenic acid 10.5 mg/kg

^b^Fe 80 mg, Cu 8 mg, Mn 80 mg, Zn 60 mg, Se 0.15 mg, I 0.35 mg

### Growth performances and immune organs index

Broiler weights and feed consumption were determined by-pen at the d1, d28 and d56, to assess total feed intake (FI), body weight gain (BWG), average daily feed intake (ADFI), average daily gain (ADG), and feed conversion ratio (FCR). FCR was calculated through the following equation: FCR  =  FI/BWG. Broilers were inspected thoroughly each day in case of recording and removing any dead birds. Mortality and culling rate were calculated based on dead and culling birds. The survival rate was calculated by counting dead and culled birds.

On d56, 1 bird per replication (6 per treatment) were randomly selected for measurement of carcass characteristics after 12-h fasting. The immune organs including, spleen, thymus gland, and bursa of Fabricius were separated and weighted, respectively. The immune organs indexes were calculated as the percentage of immune organ weight to BW.

### Serum anti-oxidant and immune globulin measurement

At day 56, 5 mL of blood was harvested (feed was withdrawn before blood sampling) from the wing vein. Serum was collected through coagulation at room temperature for 30 min and centrifuged at 3000*g* for 10 min. Serum from of all samples were stored at − 20 °C until the analysis.

Blood Anti-oxidant parameters including Superoxide Dismutase (SOD), glutathione peroxidase (GSH-px), and malondialdehyde (MDA) were determined by kits-detection methods. Similarly, concentrations of serum IgM and IgG were assayed using chicken ELISA quantitation Kits. All the assay kits were acquired from Nanjing Jiancheng Biotech Company (Nanjing, Jiangsu Province, China). All measurements were operated through the AU5421 Automatic Biochemistry Analyzer (Backman-Kelt, USA) at the First Affiliated Hospital of Nanchang University.

### Morphological examination of ileum and jejunum

On d56, ileum and jejunum samples of each slaughtering birds were collected for paraffin section. Ten villus of each intestinal segment were chosen for measuring the villus height and crypt depth in a random order to avoid bias. The ratio of villus height to crypt depth was also calculated to see the development of intestinal wall.

### Cecal sampling and microbiota analysis

On d56, cecal samples were collected from one bird per replication, and dispensed into 3 non-enzymatically sterilized cryotubes. Cecal samples were quickly frozen in liquid nitrogen, and then stored at − 80 °C for further bacterial analysis. DNA from each sample was extracted using CTAB/SDS method (Aristóteles et al. 2005). DNA concentration and purity were monitored on 1% agarose gel electrophoresis (Guo et al. 2018). The 16S rRNA gene V4 region was amplified using primer pairs F515 and R806, (F: GTGCCAGCMGCCGCGGTAA and R: GGACTACVSGGGTATCTAAT) (Gungor et al. 2016), and the detailed 16S rRNA gene sequencing program and taxonomy methods have been well documented in our pervious study (Xue et al. 2020).

Based on the taxonomy results, sequences with  >  97% similarity were assigned to the same operational taxonomic units (OTUs) (Xue et al. 2019). Following, the Green Gene Database (http://greengenes.secondgenome.com.) was applied based on RDP classifier algorithm to annotate taxonomic information for each representative sequence. Alpha diversity and beta diversity were all examined based on OTU results. All indices in our samples were calculated with Quantitative Insights into Microbial Ecology QIIME (Version 1.7.0) and displayed with R software (Version 3.15.3, R Core Team, Vienna, Austria).

### Statistical analysis

Differential analyses on growth performances and immune organs were verified through a normal distribution test using SAS (SAS Institute, Inc., Cary, NC, USA) procedure “proc univariate data  =  test normal”. Subsequently, a one-way ANOVA S–N–K test was applied to investigate the differences among the three treatments, and results were presented as mean  ±  SEM. OTUs abundances of cecal bacteria were conducted with a transformation of normal distribution using log2, and then a one-way ANOVA S–N–K test of SAS 9.2 was applied for the differential analysis. Principle coordinate analysis (PCoA) analysis was constructed using the WGCNA package, stat packages and the ggplot2 package in R software (Version 3.15.3). Spearman correlations between production performances, immune organ indexes and bacteria communities were assessed using the PROC CORR procedure of SAS 9.2 and then a correlation matrix was created and visualized in a heatmap format using R software (Version 3.15.3).For all differential analysis results, *P *value  <  0.05 was considered to be significant and 0.05  ≤  *P*  <  0.10 was considered as a tendency.

## Results

### Effects of kudzu-leaf flavonoids supplement on growth performances, immune organs indexes, and serum immune globulin content

The differential analysis of KLF supplement on growth performances were first evaluated including ADFI, BWG, ADG, FCR. Just as shown in Table 2, FCR showed a decreasing trend after KLF supplement compared with AGP in the starter phase, and compared with the CON in the finisher phase(0.05  <  *P*  <  0.1). Meanwhile, the FCR in KLF was significantly lower than that in CON treatment summarized the whole phase (*P * <  0.05). No significant differences were observed of BWG, ADG and ADFI among the three treatments, however chickens in CON treatment ate the most during the feeding phase.

**Table 2:** Effects of kudzu-leaf flavonoids supplement on the growth performances of Yellow-feathered chicken

Items		CON	KLF	AGP	SEM	*P*-value
Starter phase	BWG(g)	484.10	483.10	466.50	14.57	0.505
	ADFI(g)	27.90	26.90	27.10	0.35	0.165
	ADG(g)	17.29	17.25	16.66	0.25	0.127
	FCR	1.86	1.93	2.02	0.05	0.059
Finisher phase	BWG(g)	1084.10	1160.80	1120.70	18.18	0.269
	ADFI(g)	90.99	91.62	90.86	1.11	0.526
	ADG(g)	38.72	41.46	40.03	0.54	0.205
	FCR	2.35	2.21	2.27	0.08	0.052
Whole phase	BWG(g)	1568.20	1643.90	1587.20	32.75	0.301
	ADFI(g)	118.89	118.52	117.96	1.46	0.421
	ADG(g)	56.01	58.71	56.69	0.37	0.264
	FCR	2.12^a^	2.02^b^	2.08^ab^	0.04	0.043

*FI* feed intake; *BWG* body weight gain; *FCR* feed conversion ratio; *CON* control treatment; *KLF* kudzu-leaf flavonoids supplement treatment; *AGP* the antibiotic supplement (Aureomycin) treatments

^a,b^means within a row, different letters differed significantly (*P* < 0.05)

Immune organs were collected and weighed at the end of feeding stage, and the immune organ indexes were calculated, subsequently. Based on the results shown in Table 3, no differences were found of all immune organ indexes in both growing and finishing phases. Besides, a large standard deviation was found for both IgM and IgG in all the three treatments. IgM and IgG in both KLF and AGP treatments increased compared with CON, however, not significantly.

**Table 3:** Effects of kudzu-leaf flavonoids supplement on the immune organs and immune globulin content of Yellow-feathered chicken

Items	CON	KLF	AGP	SEM	*P *value
Liver (g)	16.48	17.29	18.58	0.35	0.169
Spleen (%)	0.14	0.12	0.14	0.015	0.736
Bursa of Fabricius (%)	0.19	0.15	0.16	0.021	0.789
Thymus (%)	0.39	0.36	0.39	0.027	0.846
IgM (ng/ml)	147.8	153.6	151.7	14.6	0.237
IgG (ng/ml)	264.4	271.3	274.8	17.8	0.467

*KLF* kudzu-leaf flavonoids supplement treatment; *AGP* the antibiotic supplement (Aureomycin) treatments

### Anti-oxidant capacity evaluation

**Fig. 1:**
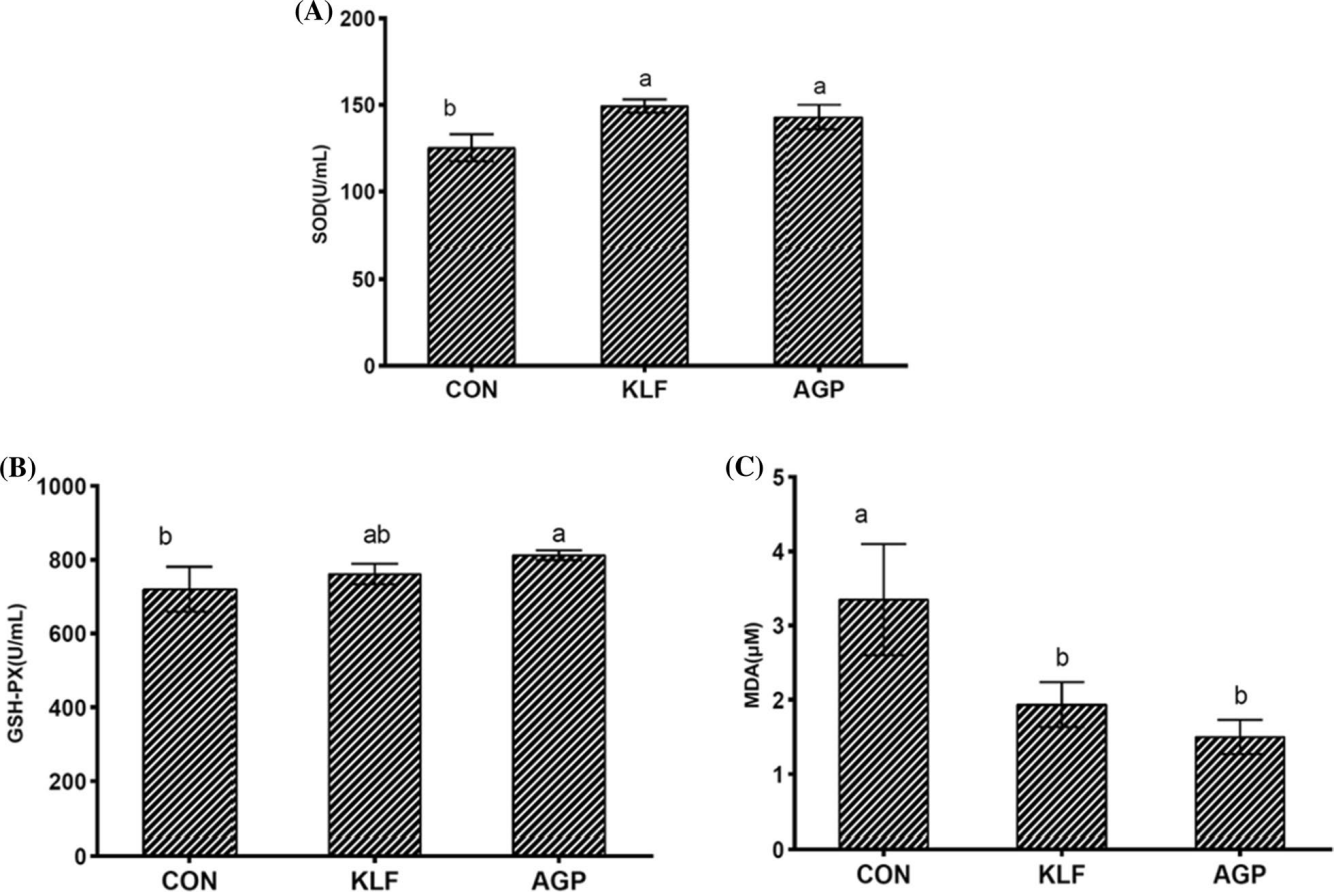
Serum Anti-oxidant capacity evaluation of CON, KLF, and AGP treatments. **A** superoxide dismutase (SOD) activity evaluation of CON, KLF, and AGP treatments. **B** glutathione peroxidase (GSH-PX) activity evaluation of CON, KLF, and AGP treatmentsC, malondialdehyde (MDA) content evaluation of CON, KLF, and AGP treatments. *CON *control treatment; *KLF *kudzu-leaf flavonoids supplement treatment, and *AGP *the antibiotic supplement (Aureomycin) treatments

Serum anti-oxidant capacities were then evaluated to investigate the protecting effects of KLF supplement on chickens. Superoxide dismutase (SOD), and glutathione peroxidase (GSH-PX) activities were measured while the malondialdehyde (MDA) content was also evaluated, and the results were showed in Fig. 1. Compared with control treatment, activity of SOD was significantly promoted after KLF supplemented, and a tendency was showed in increasing the GSH-PX activity. The MDA content was significantly decreased after KLF supplemented. No significant differences were detected between KLF and AGP treatments.

### Intestinal morphology evaluation

Morphology analysis based on the paraffin sections of ileum and jejunum were subsequently measured to investigate the supplement of KLF on the health and development of intestine. Results are shown in Table 4. Villus height of both ileum and jejunum were higher in KLF supplement treatment compared with the other two, while the Crypt depth showed a lower alteration after KLF supplement cpmpared with CON. The ratio of villus/crypt significantly increased of ileum in KLF treatment (*P * <  0.05). No significant changes were found for other items.

**Table 4:** Effect of Kudzu-leaf Flavonoids supplement on jejunum and ileum morphology

Items		CON	KLF	AGP	SEM	*P* value
Ileum	Villus height (μm)	647.4	654.3	641.0	23.246	0.526
	Crypt depth (μm)	82.84	77.81	78.98	11.314	0.355
	Thickness (μm)	137.3	145.1	126.9	67.675	0.534
	Villus/crypt	7.81^b^	8.41^a^	8.12^ab^	0.242	0.043
Jejunum	Villus height (μm)	804.4	808.5	797.4	15.34	0.517
	Crypt depth (μm)	95.32	91.73	91.01	6.21	0.198
	Thickness (μm)	155.9	158.1	156.9	6.32	0.783
	Villus/crypt	8.44	8.81	8.76	0.47	0.183

*CON* the control treatment; *AGP* the Aureomycin supplementation treatment; *KLF* Kudzu-leaf flavonoids

^a,b^means within a row, different letters differed significantly (*P* < 0.05)

### Effects of kudzu-leaf flavonoids supplement on gastrointestinal bacteria community

Relative abundances and potential function analysis on cecal bacteria of each samples in different treatments were conducted based on the taxonomy results of all samples, and these results are shown in Additional file 1. To simply state, 19 phyla and more than 250 genera were identified in the present study, and all these bacteria were used for further diversity analysis.

#### α-diversity

Alpha diversity was first applied in analyzing the internal complexity of species diversity of each treatment, and these results are shown in Table 5. In general, bacterial species in CON and KLF treatments showed a higher complexity than that in AGP, which indicated the anti-microbial functions of anti-biotics. Particularly, Shannon index performed a significant decrease in AGP treatment than those in CON and KLF treatments (*P * <  0.05). No changes were found between CON and KLF (*P * >  0.05). Besides, ACE, Chao1, and Observed species indexes showed the highest scores in KLF treatment than the other two treatments, though not significantly.

**Table 5:** Effects of Kudzu-leaf flavonoids supplement on α- diversity of cecal contents bacterial communities

Items	CON	KLF	AGP	SEM	P-value
Shannon	5.88^a^	5.81^a^	5.30^b^	0.09	0.005
Simpson	0.93	0.94	0.92	0.001	0.074
Ace	877.1	912.7	742.7	37.7	0.152
Chao1	875.7	920.6	753.5	37.0	0.164
Observed_species	742.5	752.3	613.7	30.6	0.114

*KLF* kudzu-leaf flavonoids supplement treatment; *AGP* the antibiotic supplement (Aureomycin) treatments; *SEM *standard error of the mean; *CON *control treatment

^a,b^Within a row with different letters differed significantly (P  <  0.05)

#### β-diversity

**Fig. 2:**
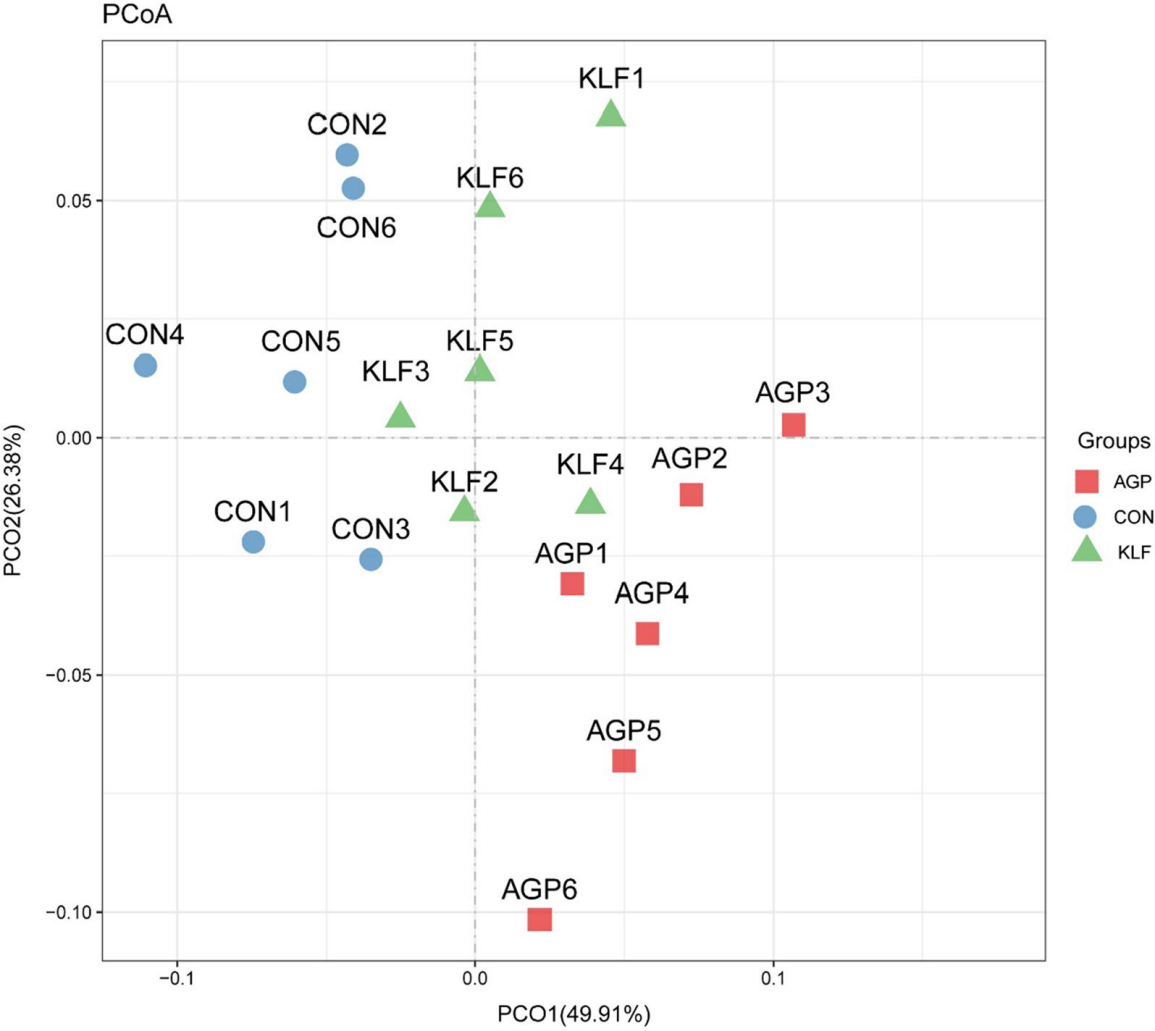
Principal coordinate analysis (PCoA) on community structures of the cecal microbiota in the different treatments. *CON *control treatment; *KLF *kudzu-leaf flavonoids supplement treatment, and *AGP *the antibiotic supplement (Aureomycin) treatment

Differential analyses on cecal bacteria of each treatment were subsequently applied and presented through PCoA. As shown in Fig. 2, PCoA axes 1 and 2 accounted for 49.91% and 26.38% of the total variation, respectively. Based on the results, bacteria communities in KLF, AGP and CON treatments could be clearly separated from each other by PCo1 and PCo2.

Differential analysis on the relative abundances of cecal bacteria at the phyla and genera levels were performed to investigate the effects of KLF supplement on gastrointestinal micro-ecosystem. Results are shown in Tables 6, 7, respectively. Among all phyla, *Bacteroidetes, Firmicutes*, and *Proteobacteria* accounted for the most 3 abundant phyla, which contributed to more than 95% of the total microbiota, and *Bacteroidetes* represented the dominant community. Relative abundance of *Bacteroidetes* in both CON and KLF, were significantly increased than that in AGP (*P * <  0.05). Besides, Proteobacteria showed a significantly proliferation after KLF supplement when compared with CON (*P * <  0.05). Whereas, the abundance was also significantly lower than in AGP treatment (*P * <  0.05). No significant changes were found on the other phyla among CON, KLF, and AGP treatments.

**Table 6:** Effects of kudzu-leaf flavonoids supplement on the relative abundances of cecal microbiota at the level of phyla

Phyla	CON	KLF	AGP	SEM	*P *Value
*Bacteroidetes*	14.97^a^	14.84^a^	14.57^b^	0.05	0.017
*Firmicutes*	14.09	14.24	14.19	0.04	0.281
*Proteobacteria*	11.09^c^	11.76^b^	12.46^a^	0.13	0.001
*Tenericutes*	7.44	7.02	6.95	0.16	0.253
*Actinobacteria*	7.66	7.40	8.37	0.27	0.222
*Elusimicrobia*	6.40	6.61	6.45	0.32	0.478
*Synergistetes*	7.26	7.17	8.11	0.15	0.098
*Verrucomicrobia*	5.83	5.59	6.00	0.21	0.871
Others	7.46	8.58	7.98	0.26	0.214

Sequences relative abundances were transformed using log2

*SEM* standard error of the mean; *CON* control treatment; *KLF* kudzu-leaf flavonoids supplement treatment; *AGP* the antibiotic supplement (Aureomycin) treatments

^a,b^Within rows and with different letters differed significantly (P  <  0.05)

**Table 7:** Effects of kudzu-leaf flavonoids supplement on the relative abundances of cecal microbiota at the level of genera

Genera	CON	KLF	AGP	SEM	P-value
*Bacteroides*	14.27	14.11	14.00	0.06	0.179
*Campylobacter*	9.50^a^	5.52^b^	4.74^b^	0.66	0.001
*Bifidobacterium*	4.74	6.13	4.30	0.34	0.066
*Butyricimonas*	8.56	8.80	8.26	0.14	0.330
*Coprococcus*	8.65	8.63	8.22	0.11	0.204
*Clostridium*	5.08	5.55	4.74	0.20	0.278
*Faecalibacterium*	10.36	9.46	10.24	0.22	0.203
*Helicobacter*	6.75^b^	9.42^a^	10.30^a^	0.49	0.002
*Lactobacillus*	7.42	7.45	6.50	0.19	0.053
*Megamonas*	8.31	9.76	7.46	0.47	0.127
*Methanobrevibacter*	5.45	6.38	4.91	0.68	0.696
*Oscillospira*	11.02	10.85	10.54	0.10	0.127
*Parabacteroides*	10.88	12.00	11.65	0.23	0.120
*Phascolarctobacterium*	9.37^b^	9.59^b^	11.05^a^	0.23	0.001
*Ruminococcus*	11.18	10.90	11.11	0.09	0.402
*Sutterella*	10.16	9.62	10.30	0.13	0.068
*Streptococcus*	3.51^a^	4.34^a^	2.47^b^	0.27	0.008
Others	13.35	12.96	13.14	0.07	0.057

Sequences relative abundances were transformed using log2

*SEM* standard error of the mean; *CON* control treatment; *KLF* kudzu-leaf flavonoids supplement treatment; *AGP* the antibiotic supplement (Aureomycin) treatments

^a,b^Within rows and with different letters differed significantly (P  <  0.05)

At the genera level, *Bacteroides, Ruminococcus, Oscillospira, Faecalibacterium*, and *Parabacteroides* accounted for the most 5 abundant genera in all the treatments. Compared with CON, KLF supplement significantly increased the abundance of *Campylobacter*, while significantly decreased *Helicobacter *(*P*  <  0.05). Furtherly, KLF also showed a significant suppressing effect on *Phascolarctobacterium*, and a significant promoting effect on Streptococcus when compared with AGP (*P * <  0.05). No other significant changes were detected among other genera for the three treatments. Particularly, probiotics such as *Bifidobacterium, Streptococcus,* and *Lactobacillus* showed the highest abundance after KLF supplement compared with the other two treatments, which might give an evidential support for the antibiotic alternative functions of KLF.

### Correlation analysis between production performances, immune organs indexes and bacteria communities

**Fig. 3:**
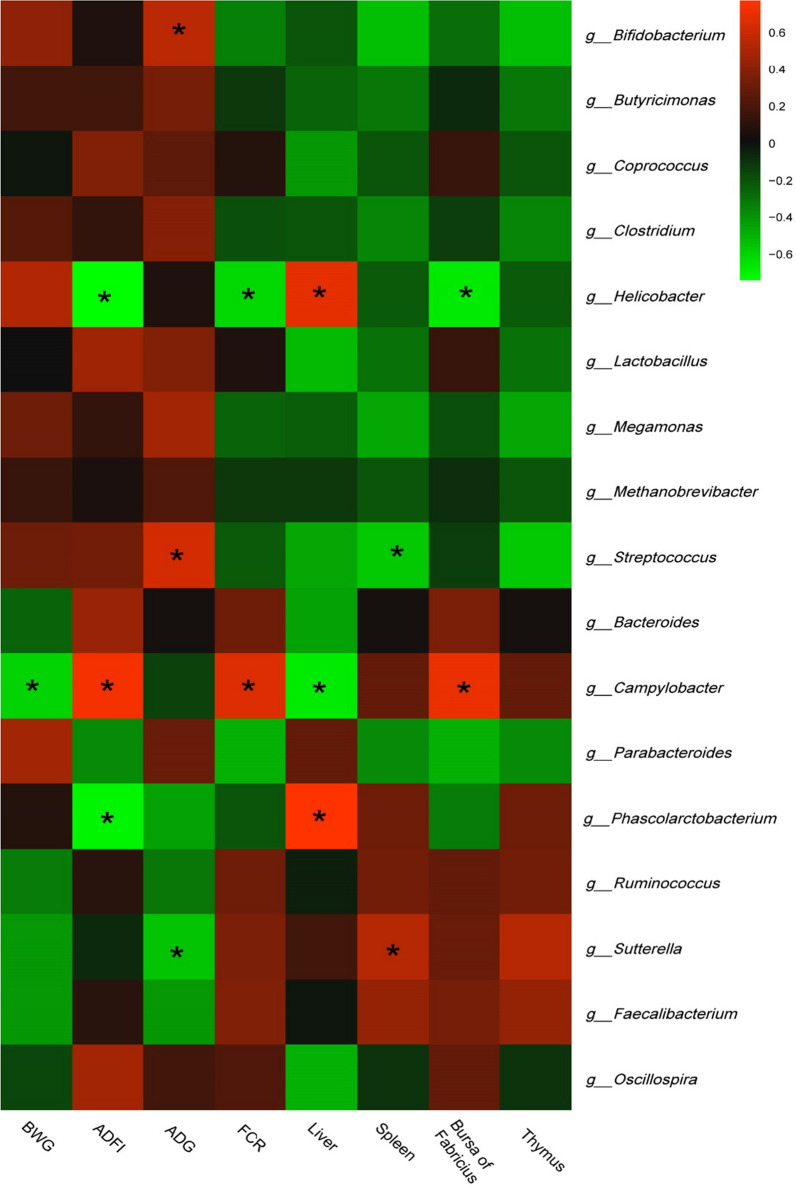
Correlation analyses between relative abundances of cecal bacteria and growth performances, and immune organs at the level of genera. The red color represents positive correlation while the green color represents a negative correlation. *A significant correlation (|r| >  0.55, *P*  <  0.05)

Correlation analysis between broiler production performance, immune organs and the most abundant bacteria communities were finally applied for investigating the effects of cecal bacteria on productions. Based on the results shown in Fig. 3, bacteria gathered into two big clusters. One was positively correlated with production performances while negatively correlated with immune organs, which included *Bifidobacterium, Butyricimonas, Lactobacillus, and Streptococcus.* The other cluster included *ruminococcus, Sutterella, Faecalibacterium, and Phascolarctobacterium,* which showed an inverse correlation with production performances and immune organs. To detailed state, *Helicobacter* was positively correlated with liver weight, while negatively correlated with ADFI, FCR and bursa of Fabrieius; *Campylobacter* showed an inverse correlation compared with *Helicobacter,* which was positively correlated ADFI, bursa of Fabrieius and FCR, and negative correlated with BWG, and liver weight. *Phascolarctobacterium* performed a negative correlation with ADFI, and a positive correlation with Liver. *Sutterella* was negatively correlated with ADG, while positively correlated with spleen*.* Particularly, probiotics including *Bifidobacterium, Lactobacillus and Streptococcus* showed positively correlated with ADG, while negatively correlated with immune organ indexes.

## Discussion

### Effects of kudzu-leaf flavonoids supplementation on production performances of broilers

Over the past few years, antibiotic alternatives including plant extract, probiotics and antimicrobial peptides were frequently investigated (Miles et al. 2006). Flavonoids showed the splendid alternative capacity owing to the powerful anti-oxidation and free radical scavenging capabilities, coupled with its easy acquisition property (Claudie et al. 2009; Wasch et al. 1998). In the present study, the KLF supplement showed a significant decrease on the FCR compared with the CON, the regulatory ability on gastrointestinal microbiome might be the key factor that could explain the increased feed efficiency.

Traditionally, gastrointestinal digestibility was mainly regulated by the composition of diets and the degrading ability of intestinal bacteria (Apajalahti et al. 2016; Saki and Alipana 2005). The microbiota in the cecum express high metabolic-activity which provided a more efficient intestinal digestibility (Xu et al. 2016) and feed utilization. KLF supplement significantly increased gut flavonoids content, which inhibited the colonization of pathogens (Hovorkova et al. 2018) and further pormoted the proliferation of bacterial, thus aroused available substrates for gut microbiota (Ohimain and Ofongo 2012), and finally increased feed efficiency.

The increasing relative abundances of *Firmicutes* also contributed to the increasing of ADG and feed utilization. Previous study has been well elaborated that *Firmicutes* provided more starch-degrading bacteria, which convert more starch into volatile fatty acids, and provided more energy and substrates for nutrients synthesis and animal growth (Barczynska et al. 2016). Besides, the ratio of *Bacteroidetes/Firmicutes* has been shown strongly correlating with lipid metabolism (Uebanso et al. 2017), especilly negative correlated with the mRNA levels of lipogenic enzymes (Cui et al. 2013). These findings might give a support that the increasing relative abundances of Firmicutes might promote the deposition of lipid and the nutrients synthesis, and therefore increased ADG.

Moreover, relative number of probiotics, such as *Streptococcus* and *Bifidobacterium*, which were positive correlated with average daily weight gain significantly increased after KLF supplemented. Probiotics in gut positively interacted with intestinal epithelium, and enhanced the intestinal digestibility (Bishnu et al. 2019; Kim et al. 2020). The increased probiotics gave evidential supports of the promoted digestibility of chickens after KLF supplement.

### Effects of kudzu-leaf flavonoids supplement on chicken health

Serum anti-oxidant capacities reflected the environmental adaptability of broilers, and benefited both intestinal and body health (Tavarez et al. 2011). Intriguingly, the increasing flavonoids content attributes to the enhanced antioxidant capacity (Cai et al. 2006). Previous study showed the powerful anti-oxidation and free radical scavenging effects and capabilities of flavonoid compounds are mainly related to their structure, ownership of A and B benzene ring structures in flavonoids strengthen the biological activity, and therefore enhanced the anti-oxidant capacities (Seyoum et al. 2006). Besides, in broiler chickens, the addition of flavonoids increased the trans-epithelial electrical resistance and stimulated the immune system response by enhancing the phagocytic activity of monocytes (Bouayed et al. 2011). Furthermore, flavonoids exerted positive effects on intestinal barriers functions (Suzuki and Hara 2011), and thus enhanced gastrointestinal development. The enhanced barrier functions prevented invading of hazardous substrates into circulation, and thus contributed to the development of intestinal mucosa, and the enhancement of intestinal health.

Furthermore, the increasing activities of Superoxide dismutase (SOD) and glutathione peroxidase (GSH-PX), and the decreasing content of MDA indicated enhanced anti-oxidant capacity after KLF supplement. The increasing activities of SOD are in line with Russo (2010), in which SOD levels was significantly increased after anti-oxidant therapy. And meanwhile, GSH-Px activity was also found significant increase after anti-oxidant treatment, in which the GSH-Px activity in the vitamin C group was increased by 33 per cent (Aydemir et al. 2015).

Generally, SOD and GSH-Px belong to the main defense anti-oxidants that prevent the formation of new free radical species (Łuszczak et al. 2011). The increased SOD and GSH-Px also increased the physical anti-stress functions, which protected animals from stressed environment and promoted the physical health of chickens.

In summary, KLF supplement improved relative abundances of gut microbiota diversity and probiotics. These results indicated the KLF could benefit the gastrointestinal health and work as antibiotic alternative.

## Supplementary Information

**Additional file 1: Table S1.** Taxonomy results of cecal bacterial community.

## Data Availability

16S sequencing raw data has been successfully submitted to NCBI, and the BioSample Accession is SAMN19589912. Other primary data used here is provided as additional files.

## Abbreviations

SOD: Superoxide dismutase
GSH-PX: Glutathione peroxidase
CON: Control treatment
KLF: Kudzu-leaf flavonoids supplement treatment
AGP: Antibiotics supplementation treatment
MDA: Malondialdehyde
BWG: Body weight gain
FI: Feed intake
FCR: Feed conversion ratio
ADG: Average daily gain
